# Unkept promises of cognitive styles: A new look at old measurements

**DOI:** 10.1371/journal.pone.0203115

**Published:** 2018-08-28

**Authors:** Félix Cuneo, Jean-Philippe Antonietti, Christine Mohr

**Affiliations:** Institute of Psychology, University of Lausanne, Lausanne, Vaud, Switzerland; University of L’Aquila, ITALY

## Abstract

Cognitive style is thought to be a stable marker of one’s way to approach mental operations. While of wide interest over the last decades, its operationalization remains a challenge. The literature indicates that cognitive styles assessed via i) questionnaires are predicted by personality and ii) performance tests (e.g., Group Embedded Figures Test; GEFT) are related to general intelligence. In the first study, we tested the psychometric relationship between the Cognitive Style Index questionnaire (CSI) and personality inventories (NEO Five Factor Inventory; NEO-FFI, HEXACO Personality Inventory Revised; HEXACO-PI-R). In the second study, we assessed the CSI, NEO-FFI, GEFT and a general intelligence test (Raven’s Standard Progressive Matrices Test; RSMT). We found that CSI scores were largely predicted by personality and that CSI was uncorrelated with GEFT performance. Instead, better performance on the GEFT was associated with better performance on the RSMT. We conclude that i) cognitive style questionnaires overlap with personality inventories, ii) cognitive style performance tests do not measure cognitive styles and should not be used as such and iii) the cognitive style concept needs to be assessed with alternative measurement types. We discuss possible future directions.

## Introduction

Much of human behaviour is assessed with reference to overall performance levels, such as being the fastest or the most accurate. Real-life requirements are, however, often more complex. For some tasks, being fast might be advantageous, while for others it might be best to stop and think. Moreover, more than one strategy might lead to the same desirable outcome, in particular in applied contexts (e.g. work output, problem solving). It is probably because of this dependency on context and outcome that a seminal publication in 1948 by Witkin and Asch [1] on cognitive styles gained influence still measurable today. These authors described that individuals differ in the way they perceive, conceptualize and/or solve problems. The resulting differences in cognitive strategy would manifest in different cognitive styles, namely in a more intuitive (field dependent) or analytical (field independent) styles. These styles were considered promising for two reasons. Firstly, they could bridge between personality and intelligence research [2–4] and, secondly, overcome controversial, hierarchical assumptions about intelligence [2,3,5]. These assumptions postulate that an intelligent individual will always outperform a less intelligent individual. In the case of cognitive styles, however, one should not be superior to the other. One style can, instead, yield a better adaptive value than the other style depending on the situation and requirements.

Despite these promises, research on cognitive styles has experienced ups and downs. In the 1970s, research activities declined, likely because a coherent theory was lacking [2]. Specifically, it has been pointed out that the field of cognitive styles did not agree on a theory that unified different methodological approaches and respective empirical results, leading to considerable confusion within the field [6]. For instance, Riding and Cheema [7] regretted that researchers in cognitive styles failed to take each other’s work sufficiently into consideration. Sternberg and Grigorenko [4] highlighted theoretical and validity problems. More recently, Evans and Waring [8] reported on 84 different cognitive style models. It is, thus, highly unlikely that researchers apply a comparable operationalization of cognitive styles making the comparability between studies difficult as well as the consideration of others’ work.

To confront these issues, researchers took two distinct ways. One way aims to cluster cognitive styles by regrouping them and the other way arranges different cognitive styles on a hierarchical model. An influential example of the first way is the suggestion by Riding and Cheema [7]. These authors regrouped cognitive styles into two orthogonal clusters. They proposed a holistic-analytic cluster representing an individual’s tendency to process information holistically or sequentially [9] and a verbalizer-imager cluster which represents an individual’s tendency to represent information verbally or through mental imagery [9]. These two clusters remain popular today [10,11]. The holistic-analytic cluster, which is of main interest to the present study, is essentially composed with the field dependence-independence dimension [12]. This dimension distinguishes individuals based on their ability to perceive an element as separate from its context and to adopt an analytical attitude in problem solving [13,14], showing an analytical-over-intuitive cognitive style [6,9,15]. Allinson and Hayes [15] proposed that the analytic cognitive style refers to logic, sequential thinking and attention to details. At the same time, the intuitive cognitive style refers to synthesis, simultaneous thinking, and attention to the whole [15].

With regard to the other way, Kozhevnikov and colleagues [2,16] argued for a hierarchical model of cognitive styles. The authors based their model on the previous work by Nosal [as cited in 16]. The general idea is that a cognitive style can be allocated to a family (or a cluster) as also proposed by Riding and Cheema [7]. In addition, for each family of cognitive styles, the authors suggest different, hierarchically-organised levels of information processing (i.e. perception, concept formation, higher-order cognitive processing, metacognitive processing). For instance, the field dependence-independence dimension belongs to the family “context dependence-independence” and to “perception” at the level of information processing. While analysis and intuition are modelled in the family “rule-based vs intuitive processing” at the level of “higher-order cognitive processing” [16].

Whether focussing on one or the other way, one challenge concerns them alike: the measurement. Cognitive styles are commonly assessed using (1) maximum performance tests (first-generation cognitive styles tests) or (2) self-report questionnaires (second-generation cognitive styles tests, mostly used today) [5,17,18]. In maximum performance tests, a performer aims to achieve the highest possible accuracy, often as quickly as possible [19]. Classically, performance tests were introduced as intelligence tests. Examples are the Embedded Figures Test (EFT) and its derivative, the Group Embedded Figures Test (GEFT) [20] as well as the Matching Familiar Figures Test (MFFT) [21]. In the EFT/GEFT, participants have to find a simple geometrical figure in a more complex pattern. In the MFFT, participants have to determine which of eight pictures matches a target picture. Commonly, in performance tests, the accurate and fast participant will obtain a high score. Yet, when linked to cognitive style, higher scores represent an analytical, or in other terms “field-independent” cognitive style [7] and lower scores represent an intuitive, or a “field-dependant” cognitive style [9]. With this type of classification, individuals who score low on spatial ability, motivation, or attention, would yield low EFT scores, and consequently would automatically be categorized as intuitive, or field-dependent persons. Kozhevnikov et al. [16] suggested that such cognitive style tests might test spatial ability rather than cognitive style. Independent studies also showed that enhanced spatial abilities or general intelligence relate to analytical cognitive style [22–25]. The results of these studies, which were reported several decades ago, remain true: such cognitive style measures seem to assess intelligence more than cognitive styles. This result is often overlooked, even nowadays (e.g. [26]).

Self-report questionnaires commonly measure states such as depression or anxiety [27] or traits such as personality [28]. With respect to self-report cognitive style questionnaires (reviewed in [17,18]), the most frequently used ones are the Cognitive Style Index (CSI), [15], the Cognitive Style Indicator [29], the Kirton Adaptation-Innovation (KAI) scale [30], the Thinking Styles Inventory [31] and most recently a newly suggested “12-scale questionnaire” [32]. In these cognitive style questionnaires, trait and state features are combined as if the aim is the measurement of a “personality in action”. By definition, personality questionnaires should assess enduring, general traits [28,33]. Cognitive style questionnaires appear to assess the consequence of such personality traits in real-life situations (e.g. work, preparing a task). For example, one personality item in the NEO-PI-R [28] reads “I am not a very methodical person”. An item in the CSI [15] reads “I find detailed, methodical work satisfying”. An item in the Cognitive Style Indicator [29] reads "A good task is a well-prepared task”. To us, both cognitive style questionnaires items seem to capture how what is assessed by this NEO-FFI item is likely expressed and experienced in concrete situations. Personality and cognitive style questionnaires might not differ much in what they assess. Von Wittich and Antonakis [34] tested, indeed, whether a cognitive style questionnaire (KAI) measures something different from an established personality questionnaire (i.e. the NEO-PI-R). Given their results, the authors concluded that i) KAI scores are well predicted by personality, and ii) the KAI has no additional explanatory value.

Returning now to the overall problem of a unique theory for cognitive styles and respective measurements, we would like to highlight a basic requirement to test theory, namely, validity of measurements. Cognitive style measures should have a high or at least satisfactory construct validity [35]: measuring what they are intended to measure. In the present study, we specifically focused on discriminant validity (i.e. the task / questionnaire does not correlate with another measure it is not supposed to correlate with). Indeed, a measure can be considered invalid if it correlates highly with another construct from which it is considered to differ [36]. Potentially, cognitive style performance measures [see 24] as well as cognitive style questionnaires [see 34] do not meet discriminant validity.

We tested the discriminant validity of the CSI [15] and the GEFT [37]. In a methodological review, Cools et al. [17] reported that the CSI was used in 22% of the studies, and is, thus, the second most frequently used instrument after the KAI [30]. The KAI is mostly used in business and management [18]. The CSI targets cognitive styles more widely [15,18]. The GEFT, the “archetypical” cognitive style performance test, is still frequently used today (e.g.[26,38]). Thus, the CSI and the GEFT seemed most appropriate to test in one comprehensive article the unkept promises of the theory on cognitive style.

In the first study, undergraduate students completed the CSI and one of two different personality inventories. Sample 1 completed the French version of the NEO-FFI [39], and sample 2 completed the French version of the HEXACO-PI-R [33]. This study aimed to replicate and generalize von Wittich and Antonakis [34] results stating that cognitive styles can be reduced to personality. We predicted that a large proportion of the CSI variance would be predicted by personality scores (see [34]). In the second study, a new student sample completed the CSI, the NEO-FFI, performed the GEFT [37] and a shortened version of the Raven’s Standard Progressive Matrices Test (RSMT) [40]. We expected that (1) CSI scores would be primarily predicted by personality scores, (2) CSI scores would be unrelated to GEFT performance, and (3) GEFT performance would correlate with performance in the RSMT. If confirmed, the second hypothesis would suggest an inconsistency in the holistic-analytic cluster (e.g. [10,11,41]) since both the GEFT and the CSI are believed to measure the same construct. Moreover, a confirmation of hypotheses 1 and 3 would support that cognitive styles are confounded with personality and / or intelligence.

## Study 1

### Method

#### Participants

Study 1 consisted of two samples of university students. All students were fluent French speakers. Sample 1 consisted of 242 students (150 females) with a mean age of 23.28 years (standard deviation (SD) = 1.87, range 20–30). They were recruited via word of mouth at the local university.

Sample 2 consisted of 336 students (271 females) with a mean age of 21.78 years (SD = 4.07, range 16–51). This sample was recruited from first year psychology students, who participated for a course credit.

#### Instruments

The original CSI [16] is a 38-items self-report questionnaire in English. Participants have to give their responses on a 3-point scale (true, do not know, false). Each item is coded 0, 1 or 2 such that higher scores indicate analytical thinking and lower scores intuitive thinking. The highest analytical cognitive style would correspond to a score of 76 and an extreme intuitive cognitive style of 0. Example items are “When making a decision, I take my time and thoroughly consider all relevant factors” (answering “true” indicates analytic-over-intuitive and scores 2) and “I make many of my decisions on the basis of intuition” (answering “true” answer indicates intuitive-over-analytic and scores 0). Normative values can be found in Allinson and Hayes’ manual [16]. We used our French translation. First, an English-French bilingual person translated the scale into French. Subsequently, the French version was back translated by another bilingual person who has not seen the original English version. The original and back translated versions were compared. Inconsistencies in meaning were discussed with an additional, proficient French-English speaker until agreement was reached.

The NEO-FFI [39] is a widely used 60-item self-report questionnaire based on the BIG-5 model of personality [28]. Each personality dimension (Neuroticism, Extraversion, Openness, Agreeableness and Conscientiousness) is assessed using 12 items. Participants give their responses on a 5-point Likert scale (from “strongly disagree” to “strongly agree”). Normative values for the French version are provided in Rolland et al. [39].

The HEXACO-PI-R [33] is a 60-item self-report questionnaire using a 5-point Likert scale (Strongly disagree–disagree–neutral–agree–strongly agree). It is a personality inventory that assesses the following dimensions of personality (10 items per dimension): Agreeableness, Conscientiousness, Emotionality, Extraversion, Honesty and Openness. Normative values are reported in Ashton and Lee [33].

#### Procedure

For sample 1, participants were given an online link. On the first page, the study was described. On the subsequent page, participants completed an informed consent form including their rights in accordance with the guidelines of the Helsinki Declaration. In Switzerland, such questionnaire research does not require further committee approval. Participants were informed that i) their answers would be treated anonymously, and ii) they could unconditionally stop the participation. Consenting participants first completed the CSI followed by the NEO-FFI. Demographic information was collected afterwards. The first author (FC) oversaw the distribution of the link with additional psychology students participating in a methodology course.

For sample 2, the procedure was the same, but they completed several additional questionnaires not relevant to the current research question of this study and will be published elsewhere. Instead of the NEO-FFI, participants completed the HEXACO-PI-R. Also, unlike sample 1, answering all questions was mandatory.

#### Data cleaning

Since participants were assessed online, it was important to consider eventual insufficient effort responders [42,43]. For this purpose, we used the personal reliability estimation [44] which consists of splitting the data in two equivalent halves per participant, with an expectation for two resulting parallel vectors of the same factors. When correlating the two vectors, we can distinguish those of low from high personal consistency. We used the 0.3 criterion [45] and excluded participants with a value under this threshold. In sample 1, 25 participants (10% of sample) were excluded, yielding a sample size of 217. In sample 2, 122 participants (33% of sample) were excluded, yielding a sample size of 214. This difference of exclusion proportion seems at first disconcerting, but at second glance reassuring. The first sample was recruited via personal contact, while the second sample was recruited via an experimental hour scheme. Likely, participants of the second sample felt less motivated to complete the questionnaires, leading to potentially random and unreliable responding occurring more frequently in sample 2 as compared to sample 1. These different attitudes might explain why more participants were excluded in sample 2 than in sample 1. According to this explanation, the current personal reliability cleaning procedure seems efficient in adhering to its goals.

#### Data analysis

First, we used the heterotrait-monotrait ratio matrix (HTMT) method [46] to assess if discriminant validity is supported by the data. The HTMT method relies on a ratio of the average correlations between constructs and the average item correlations within the same construct [47]. This method tests whether the CSI shows discriminant validity with respect to each of the five personality dimensions (sample 1) or the six personality dimensions (sample 2). To ascertain if there is discriminant validity, we applied the suggested cut-off value of .85 [47] to the ratios resulting from the HTMT analysis.

Second, we tested to which extent analytical-over-intuitive CSI scores could be predicted by personality. Importantly, if discriminant validity of the CSI with isolated personality dimensions is assessed, it still does not mean that the CSI adds relevant information to personality (see also [34]). For each sample, we performed multiple linear regression models with the personality scores as predictor variables. Sex (dummy coded) and age were entered as control variables. The conditions to perform regression analyses (i.e. linearity, normality, heteroscedasticity and outliers) were satisfied for both samples. In sample 1, we had less than 1% missing values. These were imputed using the missforest algorithm [48]. In order to *test* for validity [see 49], we assured that the resultant model is the most representative. For this purpose, we tested for two models with different features. When testing for the first model (basic model n°1), we performed a common, simple multiple linear regression analysis. When testing for the second model, we applied the Errors-In-Variables (EIV model n°2) procedure. The EIV procedure assesses the endogeneity of predictors and allows the estimation of the predictors’ true coefficients [49]. In other words, we assumed a priori that part of the Big-5 factors variance represents spontaneous errors, estimated as 1-ω [50], that is errors uncorrelated with the predictors. This error rate leads to an underestimation of the predictors’ coefficients, which can be adjusted thanks to the EIV procedure. An alternative procedure would be Structural Equation Modelling (SEM), because it also allows to adjust for error in predictors. Yet, to remain as closely as possible to the procedure used in von Wittich and Antonakis [34], and to compare our results with theirs, we favoured the EIV procedure.

### Results

#### Descriptive statistics

In both samples, all scales showed acceptable reliability (all Cronbach’s α >.75, Cronbach, 1951). For sex differences, we found in sample 1 that men scored lower than women on extraversion (t(166) = 2.29, p = .023), agreeableness (t(200) = 2.20, p = .029), and neuroticism (t(184) = 4.27, p < .001) of the NEO-FFI. In sample 2, men as compared to women scored higher on openness (t(98) = 2.32, p = .022), agreeableness (t(85) = 3.12, p = .002), and lower on emotionality (t(92) = 7.52, p < .001) and honesty (t(88) = 3.67, p < .001) of the HEXACO-PI-R. These findings are partially consistent with Ashton and Lee [33]. For a meta-analysis on gender differences in personality we refer the reader to Feingold [51]. Moreover, in sample 1, men as compared to women had higher CSI scores (t(195) = 2.19, p = .030), representing a stronger analytical-over-intuitive cognitive style [15]. This sex difference was not found in sample 2. For more information on descriptive statistics, see S1 and S2 Tables.

#### Discriminant validity and regression analyses

The discriminant validity analysis showed that the ratios between the CSI and the NEO-FFI scores were well below the cut-off score of .85 [47] (Table 1). Similarly, the ratios were well below this cut-off score for the CSI and the HEXACO-PI-R scores (Table 2). While the conscientiousness-CSI ratio was notably high (.71) for the HEXACO-PI-R score, this ratio was still below the cut-off score of .85. Thus, we can assume CSI discriminant validity from isolated personality dimensions.

**Table 1:** The ratios of the CSI with the NEO-FFI resulting from the HTMT method.

**Measure**	1	2	3	4	5	6
1. CSI	-					
1. Neuroticism	0.32	**-**				
2. Extraversion	0.4	0.36	**-**			
3. Openness	0.43	0.29	0.4	**-**		
4. Agreeableness	0.39	0.37	0.5	0.32	**-**	
5. Conscientiousness	0.58	0.32	0.26	0.29	0.31	-

The proposed cut-off value is .85 [47], with higher ratios indicating insufficient discriminant validity.

**Table 2:** The ratios of the CSI with the HEXACO-PI-R resulting from the HTMT method.

Measures	1	2	3	4	5	6	7
1. CSI	-						
2. Honesty	0.33	**-**					
3. Emotionality	0.38	0.27	**-**				
4. Extraversion	0.38	0.27	0.3	**-**			
5. Agreeableness	0.30	0.38	0.39	0.3	**-**		
6. Conscientiousness	0.71	0.37	0.25	0.31	0.27	**-**	
7. Openness	0.36	0.32	0.25	0.27	0.32	0.32	-

The proposed cut-off value is .85 [47], with higher ratios indicating insufficient discriminant validity.

While discriminant validity of the CSI from the personality dimensions can be assumed, we evaluated to what extent the CSI added new information to overall personality scores across all factors. To that end, we performed multiple regressions. The separate multiple regression analyses showed that NEO-FFI scores as well as HEXACO-PI-R scores predicted CSI scores (Tables 3 and 4). For the NEO-FFI (Table 3), the coefficients showed that enhanced conscientiousness and agreeableness, as well as lower openness and extraversion predicted increased CSI scores (i.e. a more analytical-over-intuitive cognitive style). With respect to the HEXACO-PI-R (Table 4), a more pronounced analytical-over-intuitive cognitive style was associated with enhanced conscientiousness, introversion, and emotionality.

**Table 3:** Regression analyses for the two models with the NEO-FFI.

NEO-FFI	*Model 1 β*	*Model 1 SE*	*Model 2 β*	*Model 2 SE*	*KAI β*[34]
Neuroticism	.12	.059	.13	.067	-.44***
Extraversion	-.24***	.056	-.30***	.068	.32*
Openness	-.16**	.055	-.20**	.067	.42***
Agreeableness	.25***	.054	.30***	.066	-.62***
Conscientiousness	.45***	.056	.50***	.063	-.50***
Age	-.03	.030	-.03	.030	.07
Gender–Male	.45***	.123	.44***	.123	.16**
F	19.13***		19.13***		18.26***
(df1,df2)	(7,209)		(7,209)		(10,118)
N	217		217		129
R squared	0.391		0.534		0.67
Multiple correlation	0.625		0.731		0.82

Results show the standardized coefficient (β) and the standard errors (SE). Model 2 is the EIV model [49]. In the last column, we show the results from the EIV model reported in the publication by von Wittich and Antonakis [34]. Their results are based on the KAI.

*p < .05.

**p < .01.

***p < .001.

**Table 4:** Regression analyses for the two models with the HEXACO-PI-R.

HEXACO-PI-R	*Model 1 β*	*Model 1 SE*	*Model 2 β*	*Model 2 SE*
Agreeableness	.08	.055	.10	.068
Conscientiousness	.54***	.048	.63***	.059
Emotionality	.21***	.053	.25***	.069
Extraversion	-.27***	.047	-.31***	.058
Honesty	.05	.05	-.06	.065
Openness	-.08	.05	-.09	.063
Gender–M	.08	.143	.10	.162
Age	-.03	.054	.01	.012
F	23.80***		23.80***	
(df1,df2)	(9,204)		(9,204)	
N	214		214	
R squared	0.512		0.768	
Multiple correlation	0.716		0.876	

Results show the standardized coefficient (β) and the standard errors (SE). Model 2 is the Errors-in-Variables (EIV) model [49].

*p < .05.

**p < .01.

***p < .001.

To Table 3, we added results on the KAI (another cognitive style measure assessing innovator and adaptor cognitive styles) as published in von Wittich and Antonakis [34]. Higher KAI scores represent an innovator over adaptor cognitive styles. Results on the KAI replicated our findings, namely that NEO-FFI scores predict KAI scores. In both studies, extraversion and openness were associated with intuitive and innovator cognitive styles, while conscientiousness and agreeableness were associated with analytical and adaptor cognitive styles.

When comparing the R-squared values for the first model and the second EIV model, the values were higher when using EIV models (Tables 3 and 4). With EIV models, we obtained R-squared values i) of 0.53 when predicting CSI scores with the NEO-FFI and ii) of 0.77 when predicting CSI scores with the HEXACO-PI-R. The multiple correlation values for these two models were 0.72 and 0.88, respectively. Given that the CSI has error that is unpredictable, these multiple correlations should be compared to the reliability of the CSI (ω = 0.88 or α = .87). This reliability estimates the correlation of the CSI with the latent trait it aims to assess. The prediction quality with the NEO-FFI is slightly lower than the CSI reliability, while with the HEXACO-PI-R, the prediction quality is equal to the CSI reliability.

### Brief discussion

We tested the discriminant validity of the CSI questionnaire when compared to the NEO-FFI personality inventory [39] (sample 1) and the HEXACO-PI-R [33] (sample 2). As we collected data online, we first cleaned the data using the personal reliability method [44]. We first tested simple discriminant validity between the CSI and isolated personality dimensions with the HTMT. Then, we assessed the part of explained CSI variance using regressions with EIV modelling to control for biases from the error of explicative variables [49]. Based on previous cognitive style assessment criticisms [2,34], we expected that the frequently used CSI [15,18] might not add much information to the information provided by conventional personality questionnaires [34]. Our main findings are threefold; we found that i) the CSI showed discriminant validity from single dimensions of both the NEO-FFI and the HEXACO-PI-R, ii) the NEO-FFI and the HEXACO-PI-R importantly predicted CSI scores and iii) when testing for validity, controlling for error bias (e.g. using EIV modelling and data cleaning) improves the part of explained variance.

Regarding the first finding, as suggested by the HTMT [46] results, we suggest that the CSI cannot be reduced to a single personality trait. This is unsurprising, since the CSI was not designed to measure the same latent variables as those of the NEO-FFI or the HEXACO-PI-R.

Still, satisfactory discriminant validity does not mean that CSI scores add relevant information when used together with a personality inventory, as can be inferred from the results of the multiple regression analyses and corresponding findings (i.e. second and third findings). The second finding indicated that the proportion of CSI variance was largely explained by variance in the conventional personality inventories. Higher CSI scores (i.e. a stronger analytical-over-intuitive cognitive style) were predicted by enhanced agreeableness, and conscientiousness and by reduced extraversion and openness [see 15]. This considerable overlap in score variance for the CSI and both the NEO-FFI and the HEXACO-PI-R suggests that the intrinsic properties of cognitive styles questionnaire items are similar to those of personality inventories (e.g. analytical traits are related to facets of conscientiousness such as dutifulness and self-discipline). This observation supports results of an independent study, in which KAI scores were predicted by personality scores in a similar way [34]. Noteworthy, the CSI and KAI have been allocated to the holistic-analytic cluster [7] (i.e. the adaptor and the analytic are both described by introversion, conscientiousness, agreeableness and low openness), and scores in both can be explained to a large extent by scores in conventional personality questionnaires.

With respect to the third finding we would like to highlight that in assessing the proportion of CSI variance explained, we controlled for two biases: (1) the data could have noise (e.g. insufficient effort responding [42,43]) and therefore needs to be cleaned (e.g. [44]), and (2) predictors have errors (that might lead the model to underestimate the coefficients) and thus needs to be taken into account by methods such as EIV modelling [49] or SEM. Potentially, without correcting for these biases, the high overlap between CSI scores and personality questionnaire scores would have been missed, or worse, the lower correlations could be taken as validation proof. In fact, some researchers [3,30] expect correlations between cognitive styles and personality, but none postulated that the prediction would almost be perfect.

In sum, we found that CSI scores can be psychometrically dissociated from individual personality subscales scores. However, a combination of these traits (i.e. with a multiple regression) predicted CSI scores. The latter results on the CSI replicate a comparable finding formerly found for the KAI [34]. We suggest that the comparable results on both the CSI and KAI might generalize to further cognitive style questionnaires. Unlike von Wittich and Antonakis [34], we do not agree that cognitive styles are no longer “in style”. We propose, instead, that the questionnaire methodology does not seem appropriate to measure cognitive styles (see also general discussion).

## Study 2

Study 1 focussed on self-report cognitive style questionnaires. In the cognitive styles literature, however, performance tests are the historically older type of cognitive style measurements. To investigate whether results from questionnaires and performance tests are related in any way (in the end, they are considered to both measure the concept of “cognitive styles”), we decided to test (1) whether two “conventional” cognitive style measures correlate with each other (CSI and GEFT) and (2) whether the GEFT correlates with “another” performance measure rather than a self-report cognitive style measure (again the CSI). We used the Raven’s Standard Progressive Matrices Test (RSMT) [40] to assess “another” performance measure, because **t**he RSMT is a valid (and quick) measure of general intelligence [52]. The outcome of the current study is key in accepting the construct validity of cognitive style measures as frequently claimed in the published literature.

### Method

#### Participants

We recruited 119 students (77 females) with a mean age of 23.60 years (SD = 1.76, range 20–30) via word of mouth at the local university. All participants were fluent French speakers.

#### Instruments

As in Study 1, cognitive style was assessed with the French translation of the CSI [15] and personality with the French version of the NEO-FFI [39].

The RSMT [40] is a good and quick measure of general intelligence [52]. It consists of 60 items, which are equally distributed (n = 12 items each) across five sets (A, B, C, D, E), but of increasing difficulty. Items consist of an arrangement of black and white visual pattern in matrix form. Of the possible patterns, all are presented but one. Participants have to deduce the rule that is the common denominator to the pattern and infer the missing one. Each item is scored 1 if the missing pattern is found and 0 otherwise. Thus, RSMT scores range from 0 to a maximum score of 60. We performed a preliminary test with a student population. This test showed a ceiling effect for sets A and B. They were too easy for this population. Consequently, participants were administered the more difficult sets C, D, and E (n = 36 items) which they were required to complete within a time limit of 10 minutes.

The GEFT [37] measures the field dependence-independence dimension which is part of the holistic-analytic cluster. First, participants are briefly presented with a simple geometric figure. Then, a more complex figure is presented in which the previously presented figure is included. When presented with the complex figure, participants are instructed to find (and draw) the initial simple geometric figure. The test consists of 18 items of increasing difficulty. The participants had 12 minutes to perform the task. If the participant draws the simple geometrical figure, the item is scored 1 and 0 otherwise. Thus, scores range from 0 to a maximum score of 18.

#### Procedure

Participants received an online link. The procedure was analogous to the one for sample 1 in Study 1. Participants completed the tasks in the following order: GEFT, RSMT, CSI, and NEO-FFI.

#### Data cleaning and analysis

In line with Study 1, the sample was cleaned using personal reliability estimation [44], applying, as before, the 0.3 criterion [45]. We found and removed 12 cases of insufficient effort responders, leaving a total sample of 107 participants.

Using simple correlations, we looked at which extent CSI and GEFT scores were associated. Subsequently, we tested the correlation of these scores with the NEO-FFI and RSMT scores, respectively. We also expected that (1) the correlation between GEFT and RSMT would be larger than the correlation between GEFT and CSI and (2) the correlation between a personality dimension (here conscientiousness, as it seems to be the most related personality dimension to analytical style, see Table 3) and CSI would be larger than between GEFT and CSI. To compare correlations statistically, we used Hittner, May and Silver’s statistic [53].

### Results

All measures had acceptable reliability (all Cronbach’s α >.70). For sex differences, independent t-test showed that men were less extraverted (t(96) = 3.58, p < .001) and older than women (S3 Table). The current sample scored higher in openness and conscientiousness and lower in neuroticism than a normative French student population [39] (S3 Table). Also, GEFT scores were not normally distributed (W = 0.79, p<0.001), showing a skewness to the left of -1.71 (SE = 0.22). This implies that the GEFT suffers from a ceiling effect [see 54]. Therefore, we used Spearman’s coefficient for all correlations including the GEFT.

Three of the five personality dimensions correlated with the CSI (Table 5). CSI scores were associated with higher Agreeableness (r(105) = .237, p = .014) and Conscientiousness (r(105) = .385, p < .001), and with lower Extraversion (r(117) = -.411, p < .001). CSI did not correlate with GEFT or RSMT (Table 5), but GEFT and RSMT were significantly correlated (r_s_ = .352, p < .001). Comparing correlation coefficients led to the conclusion that the correlation between GEFT and RSMT was larger than between GEFT and CSI (z = 2.01, p = .023, one-sided) and that the correlation between CSI and conscientiousness was larger than the correlation between CSI and GEFT (z = 2.04, p = .020, one-sided).

**Table 5:** Correlation matrix.

	1	2	3	4	5	6	7	8
1. Openness	.804	-.073	.210*	.047	.240*	-.155	.074	.050
2. Conscientiousness		.878	-.095	.12	-.235*	.385***	-.033	-.139
3. Extraversion			.764	.232*	-.268*	-.411***	-.033	.069
4. Agreeableness				.821	-.126	.237*	-.100	.129
5. Neuroticism					.855	.049	.012	-.118
6. CSI						.886	.087	.099
7. RSMT							.902	.353***
8. GEFT								.912

The top-right triangle shows correlations, the diagonal shows Cronbach’s alphas, the GEFT column displays spearman correlations (not normally distributed).

*p < .05.

**p < .01.

***p < .001.

### Brief discussion

In this second study, we tested i) whether we could replicate the observed association between CSI scores and the personality questionnaire scores from Study 1, ii) whether two different cognitive style measures—a questionnaire (CSI) and a maximum performance test (GEFT)—associate with each other, and iii) whether the relationship (if any) between the CSI and GEFT would be lower than the relationship between the GEFT and another performance measure (RSMT). We replicated results from Study 1, showing that CSI scores correlated with three NEO-FFI personality dimensions. With regard to the second point, CSI scores did not correlate with GEFT scores, questioning a relationship between the various cognitive style measures (see [24]). Yet, we confirmed that GEFT performance correlated with RSMT performance, an intelligence measure [23].

We already discussed the first results in the brief discussion of Study 1 (i.e. correlations between CSI scores and personality scores). The next result to be discussed is that GEFT scores correlated with RSMT scores, but not with CSI scores. The absent correlation between CSI scores and GEFT scores questions the notion that the two measures assess the same construct [24] or refer to the same cognitive style family [7]. Thus, this result would contradict the notion that cognitive styles can be grouped into two orthogonal cluster [7], but would not contradict the assumptions of the hierarchical cognitive styles model [2,16]. This latter model postulates that analysis-intuition and field dependence-independence are different cognitive styles and would not be expected to correlate. While the interpretation of the current result depends on the theory at hand, the other results cast doubts over cognitive styles measurements.

The cognitive style concept assumes that a cognitive style should be value-free. We found, however, that higher field independent style (as assessed with the GEFT) associated with higher intelligence (as assessed with the RSMT). This result clearly goes against the idea of value-free features in cognitive styles (i.e. that a cognitive style should not be superior to another). Additionally, a bipolar concept (i.e. a cognitive style dimension such as analysis-intuition can be defined as opposite ends of the continuum: e.g. intuition is not just the absence of analysis) should not match with the observed skewed distribution of GEFT scores. We doubt that this skewness can be explained by having selected a population with a particularly high analytical cognitive style. Instead of assessing cognitive styles, it seems more likely that the GEFT represents a test of general (or spatial) intelligence, a seemingly easy task for our student population.

In the following section, we will discuss whether the results from the two studies can be integrated into the field of cognitive style, or whether the conclusion should rather be to abandon the concept of cognitive styles all together [34].

## General discussion

Cognitive styles have remained a widely studied psychological theory ever since its introduction in the 1950s, even after experiencing serious criticisms in the 1970s [2–4,7,8,13,16]. Cognitive style theory assumes that individuals have a particular way of thinking and processing information [2]. By default, such cognitive styles should not be hierarchical (one being superior to another). At the time of their conceptualization, this point of view promised that cognitive styles go beyond the hierarchical assumptions of intelligence [2,3,5,55]. While intelligence is a general indicator of an individual’s performance, the promise of cognitive styles was to allow for a more in-depth examination of how an individual adapts to a situation. In this framework, a less intelligent individual could yield a good performance if the situation matches the person’s cognitive style. Analogically, a more intelligent individual could also yield a bad performance in a situation that does not match this person’s style. This context-specific reasoning would imply that a person with a given cognitive style might be favoured in one situation but not necessarily in another situation [56]. Given the wide interest in cognitive styles, it is surprising that since the middle of the last century, and despite some attempts [2,7,16,57], there is not an all-encompassing model of cognitive styles that is widely accepted. Numerous measures exist and new ones continue to be introduced [32]. Yet, we do not know whether and how these measures relate to each other and would assess what they are intended to measure. This situation is what motivated us to perform the present studies.

In two independent studies, we tested i) whether the widely used CSI questionnaire [15] assesses “something” that is beyond what is already measured through common personality questionnaires, ii) in which way a cognitive style questionnaire (CSI) correlates with a cognitive style performance test (GEFT), and iii) whether a cognitive style performance test (GEFT) measures an ability (or intelligence) rather than a cognitive style. Results showed that despite CSI showing discriminant validity with personality dimensions, much of its variance is, indeed, explained by variance of these personality dimensions. The CSI was unrelated to GEFT performance, but GEFT performance correlated with superior performance in the RSMT, a general intelligence test [22–24,58].

These results challenge the validity of both cognitive style questionnaires and cognitive style performance tests due to the confounds with personality traits and intelligence. Firstly, two measures of the same concept (holistic-analytic cognitive styles) did not correlate with each other (CSI and GEFT). While this result underlines a more general psychometric issue where objective (performance) and subjective (questionnaire) measures of a given concept are frequently unrelated [59,60], it also suggests that at least one or both measures seem inapt to measure their intended construct. Secondly, the CSI showed discriminant validity with the single factors of two personality inventories, yet, it was highly predicted by those personality factors. This raises the question of the worrying overlap of cognitive style questionnaire constructs with the more general personality concept (see [17,18]). Thirdly, GEFT performance correlated with RSMT performance (a general intelligence test), which is inconsistent with the bipolarity of the cognitive style concept.

In the general validity literature, Campbell and Fiske [36] suggested to investigate which of the following three scenarios apply when two methods of measuring a given concept are unrelated (in the current case, the CSI and the GEFT). Firstly, neither method is adequate for measuring the concept at hand. Secondly, one of the two methods is not measuring the trait (it might be a measure of another trait). Thirdly, the trait has conceptual problems and should be developed or abandoned. Von Wittich and Antonakis [34] were in favour of the third possibility. They showed that a cognitive style questionnaire, the KAI was largely predicted by scores on a common personality questionnaire. Additionally, when adding the KAI scores to a model predicting leadership type by personality scores, the cognitive style measure (KAI) did add explanatory value. While their conclusion to abandon the concept of cognitive style seems, from their point of view justified, we would want to consider the other two possibilities too.

When considering the first (neither of the methods are adequate) or the second possibility (one of the two methods is not measuring the trait, i.e. it might measure another trait), we can “question” the CSI (questionnaire) or the GEFT (performance test), or both. GEFT performance correlated with enhanced performance in an intelligence measure, but not with the CSI or a common personality measure. Thus, one has to ask whether GEFT measures cognitive styles. Four decades ago, Weisz et al. [24] already highlighted that cognitive style researchers should aim to delineate how cognitive style and a more fundamental cognitive level (i.e. intelligence) differ. While GEFT correlated with RSMT (both are performance tests), and the questionnaires correlated amongst each other, GEFT did not correlate with the CSI or with either of the personality dimensions. Thus, it seems that cognitive style measures of either type seem inapt to fulfil the cognitive styles promises, as they do not bridge between personality and intelligence—as originally intended [2–4]—and fails to overcome hierarchical assumptions about intelligence [3]. Instead, maximum performance tests (including the GEFT) seem to represent a typical measure of intelligence and questionnaires represent a typical measure of personality. These conclusions suggest that cognitive style measures fall in the first Campbell and Fiske [36] scenario, i.e. neither questionnaires or performance tests are adequate for measuring cognitive style concept.

This conclusion would suggest that researchers should stop using maximum performance tests to assess cognitive style (such as the GEFT, e.g. [26]). Indeed, these tests seem seriously confounded by intelligence. Similarly, researchers should stop using the current cognitive style questionnaires, particularly when they largely overlap with personality (see [34]). In fact, cognitive styles are expected to display discriminant validity with personality [17,18]. Moreover, proposing new cognitive style questionnaires (e.g. [32]) might not be ideally placed, because they will likely be widely predicted by personality.

We conclude that we should focus on the development of theory-driven measures of cognitive styles that satisfy the promises of the concept. Neither cognitive style should be superior to another. Improved or new measures of cognitive style should satisfy at least two conditions that are key to the definition of the concept of cognitive style. The first condition concerns the notion that cognitive styles are “cognitive”: they emerge when a person is treating incoming or existing information. Therefore, they should be assessed and evaluated while participants perform an a priori tailored cognitive task (rather than filling in a questionnaire). The task should be sensitive to information processing that would emerge differently when triggering a person’s cognitive style. The second condition is part of the cognitive style definition, i.e. that the dimension is value-free and therefore the measure should not correlate with an intelligence measure [2,61].

One existing test satisfies both conditions, the Cognitive Style Analysis [41,62], because it consists of two performance tests (one for holistic and one for analytic cognitive style). To our knowledge, the CSA is the only test that measures positively both holistic and analytic cognitive styles. The final score is computed as the ratio between the two, indicating in which test, the participant performed better and to what extent. This ratio allows the CSA to be value-free [63] because it does not report the overall performance. Unfortunately, the CSA displays serious reliability issues [62,64,65], perhaps due to the lack of discrimination between the holistic and analytic performances tests. These issues probably account for the lack of interest from the scientific community. In fact, Cools et al. [17], in their review, found that the CSA was used in only 3% of the studies they investigated. We consider the reliability of the CSA to be an issue, yet, the attempt in creating such a test that respects the cognitive aspect of cognitive styles (unlike questionnaires) as well as their value-free feature (unlike performance tests) is a legitimate path. Further attempts should be taken to improve the CSA or introduce improved versions using a similar test design. In line with this last proposition, we argue for the development of cognitive style tests, which assess poles of cognitive style (e.g. analytical and holistic) independently. When these tests are clearly defined, strong reliability will eventually be met, and a theoretically and empirically sound measure of cognitive style would arise.

Finally, we would like to highlight some limitations of our studies. We questioned cognitive style questionnaires, and could show that their scores were well predicted by personality questionnaires. We can conclude that this prediction has now been confirmed for two questionnaires, the CSI and the KAI [34]. We suggest that similar predictions hold true for other cognitive style questionnaires. Yet, testing so would involve a huge research effort given the already high number of instruments, and the new ones that are being added [e.g. 29]. Moreover, we think it would have been beneficial to also determine an external criterion for a match of situational demands and cognitive styles. For instance, within the same professional setting (e.g. computer science), one domain requires very local and detailed processing (development) while the other domain requires global and overarching activities (e.g. system architecture). According to cognitive style theory [66], the former needs would benefit from a person with more analytical capacities, and the latter a person with more holistic capacities. If the match between situational demands and cognitive styles has not been met, job satisfaction for either person should be higher as compared to a situation of mismatch. Another limitation is the testing of student populations who do not experience the challenges and processing strategies people are faced with in other life domains. Moreover, in the first study, the second sample was composed solely of psychology students. Future studies using student populations should, at a minimum, distinguish between students from different faculties, or from different higher education domains (e.g. including architecture, the arts). Ideally, studies should target professional of different positions requiring an a priori different cognitive style.

## Conclusion

For many decades, cognitive style research experiences ups and downs for various reasons [2,7,8,31]. Here, we regrouped different criticisms on cognitive styles methodology and aimed to form some unifying conclusions and suggestions based on the outcome of our empirical studies. Our results indicate that the methodology employed in the field of cognitive styles should be reconsidered. In two independent studies, we observed that cognitive styles questionnaires were predicted by personality questionnaires, in line with recent results (see also [34]), but not by a cognitive styles performance measure. The latter, however, correlated with an intelligence task (see also [22–25]). We concluded that cognitive styles questionnaires overlap with personality and cognitive styles performance tests are actually assessing aspects of intelligence. Discussing what would be the best way moving forward, we suggest that the research community would gain much from answering the still unanswered call of Weisz et al. [24], that cognitive styles and cognitive abilities need to be systematically delineated. One suggestion is to develop two independent performance tests, one for each opposing pole of a cognitive style dimension (e.g. analytic-local vs holistic in the case of the CSA). The computation of a ratio between these two performances would be independent of intelligence and can provide an adequate measure of cognitive styles [41,62].

## Supporting information

S1 Table Descriptive statistics, study 1, sample 1. Means and SD for the demographic and questionnaire data for men (n = 92) and women (n = 150) separately (full sample). Results from sex comparisons (t-tests) and Cronbach’s alpha [67] and Omega total [50] are equally presented. Significant results are given in bold. Last columns present sample comparisons with a French student population [39].
(XLSX)

S2 Table Descriptive statistics, study 1, sample 2. Means and SD for the demographic and questionnaire data for men (n = 65) and women (n = 271) separately (full sample). Results of the sex comparison (t-tests) and Cronbach’s alphas and omegas are equally presented. Significant results are given in bold. Norms columns present p-values of the t-test comparing our men and women with normative values [33].
(XLSX)

S3 Table Descriptive statistics, study 2. Means and SD for the demographic and questionnaire data for men (n = 42) and women (n = 77) separately Results from the t-tests, Cronbach’s alphas and omegas are equally presented. Significant results are given in bold. Last columns present sample comparison with a French student population [39].
(XLSX)
